# Clueless: An ethnographic study of young men who participate in the seduction community with a focus on their psychosocial well-being and mental health

**DOI:** 10.1371/journal.pone.0229719

**Published:** 2020-02-26

**Authors:** Rob Whitley, JunWei Zhou

**Affiliations:** Department of Psychiatry, Douglas Mental Health University Institute, McGill University, Montreal, QC, Canada; Newcastle University, UNITED KINGDOM

## Abstract

In the last decade, a cross-national community of like-minded young men has emerged, commonly known as ‘the seduction community’. This community is led by professional ‘pick-up artists’ who teach these young men a variety of techniques and mindsets with the stated aim of improving their success with women, or ‘game’. There has been little research on the men who participate in this community, and none from a mental health angle. As such, this study is propelled by two specific objectives, namely documenting and understanding (i) the reasons why young men join the seduction community; and (ii) the impacts of community involvement on participants’ lives. To meet these aims, we used an inductive qualitative methodology giving ample scope for bottom-up understandings to emerge. Specifically, we recruited young men participating in the seduction community for an in-depth qualitative interview (N = 34) to explore self-reported motives and impacts. Interviews were augmented by lengthy participant observation, and data was analyzed by content analysis techniques. The results reveal that men often join the community to address a range of psychosocial deficits, and that community involvement successfully equips participants with numerous valued social and communication skills. The community appears to fill a void in providing a place of hope, fellowship and learning for young (often immigrant) men. The findings are summarized in five themes (i) loneliness and social inclusion; (ii) lack of male role models and need for guidance; (iii) mental health and well-being issues; (iv) skill acquisition and personal development; and (v) the dark side of pick-up. Interestingly, some of the practices commonly taught and utilized within the community resemble aspects of Cognitive Behavioral Therapy and mental health peer support. This may explain its evident appeal. We conclude by reflecting on the implications of the findings for official mental health service provision for young men.

## Introduction

In the last decade, a large cross-national community of like-minded young men has emerged, ostensibly dedicated to learning a variety of techniques and mindsets with the stated aim of improving their success with women, or ‘game’ [1]. This community has labelled itself ‘the seduction community’, a term also employed by academics and used in the current paper [2–6].

The seduction community is led by professional ‘pick-up artist’ (PUA) instructors who teach various aspects of ‘game’ to eager young men in the community. Leading PUA instructors were depicted in Neil Strauss’ best-selling book ‘The Game’ [7], which reached the New York Times bestseller list, and reportedly sold over 2.5 million copies [8], as well as in the 2007 TV series ‘the Pick-Up Artist’, which was shown across North America on the VH-1 channel. Viewing figures in season one were high enough to warrant a second season of ‘the Pick-Up Artist’, which was shown in 2008 [9].

The origins of the seduction community can be traced to Los Angeles in the early 2000s, when two PUA instructors known as ‘Mystery’ and ‘Tyler Durden’ began systematizing and commercializing ‘pick-up’ [7]. Since then, the community has expanded and the pick-up industry was recently estimated to be worth around 100 million USD [1], with numerous companies offering a variety of products including in-person seminars, books, individual consultations, on-line courses and ‘bootcamps’. These ‘bootcamps’ are led by a professional PUA instructor who teaches pick-up techniques to a small group of men, paying up to 3 000USD each [10]. They last around three days and involve classroom teaching as well as ‘in-field’ instruction on the streets. Most major North American and European cities regularly host well-attended ‘bootcamps’ [10,11]. These cities also frequently contain PUA ‘lairs’ (or ‘inner circles’), where young men meet together regularly to discuss and practice various aspects of game, with one website reporting around 240 ‘lair’ groups across the world [12]. These ‘lairs’ are augmented by concomitant on-line discussion groups on Facebook and other social media [13].

Indeed, the seduction community is welded together by numerous websites and internet forums. For example, one of the most famous PUAs is Julien Blanc, an in-demand instructor with Real Social Dynamics (RSD); a prominent business that is a market leader in providing PUA related services. Blanc’s YouTube channel ‘JulienHimself’ has over 140 000 subscribers and 15 million views [14]. Other leading instructors host internet forums which have thousands of participants discussing a variety of ‘game’ related threads. In a BBC documentary PUA instructor ‘Roosh V’ claimed that his website receives 1 million views a month [15]. Similarly, the seduction community has a very active sub-reddit which has over 440 000 members, with members regularly posting stories and seeking advice related to ‘game’ and pick-up artistry [16].

### PUA scandals and criticism

The 2005 publication of ‘the Game’ brought the activities of the seduction community to a wider audience. However the community generally operated under the radar of society until 2014, with journalist Amanda Marcotte noting that ‘pick-up artistry is a huge, if generally ignored industry’ [17]. However in the last five years, the community has been rocked by a number of scandals involving PUA instructors, leading to much negative media coverage and wider discussion of the community. Three examples are given below.

The first involved the aforementioned Julien Blanc, who was subject to a deplatforming campaign, based on allegations that his teachings encouraged the abuse of women. More than 77 000 people signed a petition to ‘keep Julien Blanc out of Canada’ [18], with the then immigration minister stating that Blanc’s material was ‘completely counter to Canadian values and common decency’ [19]. He was not formally banned, but the negative publicity led to the cancellation of numerous scheduled PUA events across Canada [19].

The second involved PUA instructor Roosh V who engaged in a ‘world tour’ in 2015 to give seminars on ‘the state of man’. His planned visit to Canada met with sustained opposition, due to concerns that his teachings were aggressively misogynistic and encouraged abuse of women. Over 45 000 people signed a petition to prevent his entry into the country, and the Mayors of Montreal and Toronto publicly opposed his presence [20]. This campaign failed, and Roosh V successfully held seminars in Toronto and Montreal.

The most recent involves PUA instructor ‘Addy A-Game’, who was found guilty in 2019 of threatening and abusive behaviour, including following two teenage girls (aged 16 and 17) down a secluded lane in Scotland. He was sentenced to two years in prison, amidst much negative media coverage. Indeed, his activities were brought to public attention through a BBC exposé-style documentary entitled ‘Secrets of the Seduction Bootcamp’ [21], which was shown on national prime-time television in late 2019.

### Research on the seduction community

Interestingly, there has been little academic research on the seduction community, despite its evident popularity with young men and the high number of adherents. The few studies that have occurred reveal a complex and inconsistent picture, with divergent results and a series of limitations.

For example, two studies were based on a content analysis of texts written by experienced PUA instructors. Based on their content analysis of six books written by PUA instructors, Almog and Kaplan conclude that the seduction community is ‘characterized by hyperconsumerism and objectification of women [which] culminates in the dehumanization of all parties’ [5]. Similarly, Denes analyzed a single-text written by the PUA ‘Mystery’, concluding that his formulaic seduction methods involve an ‘aggressive and coercive approach reflecting characteristics of rape scripts’ [22]. These studies have two serious limitations; they focused solely on instructors’ texts and did not involve research with ordinary men in the community.

Other studies have attempted to overcome these limitations through ethnographic research. O’Neill [3] conducted 32 interviews with a range of community leaders including freelance trainers, PUA company employees, event managers, business directors and some students. She found that ‘how to more skillfully negotiate casual sexual encounters was the immediate priority’ for men involved in the community, also noting that ‘women are not only objectified but made into object lessons’ in community teachings. This led her to conclude that the community is a ‘deeply problematic phenomena’. That said, she does note that one of the most frequently recurring narratives in the interviews is that community participation can improve men’s social skills, which led to reported benefits in their professional lives.

Interestingly, the three above-cited studies could be said to proceed from a ‘hermeneutic of suspicion’- a label given to research that is inherently suspicious of the topic under consideration [23,24]. All three studies openly adopt a pro-feminist framework and interpretive stance. Moreover, they fail to foreground the voice of the ordinary rank-and-file men who make up the bulk of seduction community members. In contrast, a few studies have proceeded from a ‘hermeneutic of faith’, attempting to give voice to men involved in the seduction community from a more neutral non-judgmental stance. This is reflected in their methodology, which involves in-depth qualitative research with ordinary rank-and-file community members.

For example, Schuurmans & Monaghan [6] conducted interviews with 22 men participating in the seduction community to understand young men’s motivation for getting involved. Like O’Neill [3] they found that the desire to become more attractive to women was a recurring theme. However they noted that ‘the sexual prospect was not the sole motivator’, and discuss several other common reasons for involvement. These include addressing issues such as social isolation, loneliness and personal development. Interestingly, many men in their sample were immigrants, who felt particularly isolated and socially unskilled within the host U.S. culture, leading them to join the community. Notably, this study found a dark-side to pick-up, highlighting its potential ‘all consuming nature’ and its costs in terms of time, money and men’s well-being.

Hendriks [4] conducted a similar study proceeding from a ‘hermeneutic of faith’, using participant observation methods, including attendance at ‘lair’ meetings and a PUA ‘bootcamp’ to better understand common discourses and practices. Interestingly, he found that common teachings often focused on self-development and personal growth, with students frequently challenged to break out of their ‘comfort zone’ through various psychosocial activities. Hendriks concludes that underneath the ostensible goal of ‘success with women’, teachings and practices in the community focus on self-improvement and personal growth through ascetic self-discipline. These nuanced findings complexify the small literature on the community.

### The social science and mental health context

The expansion of the seduction community and its growing appeal to young men is occurring within a rapidly shifting social world, especially with regards to norms surrounding gender roles and gender relations. For example, leading sociologists cogently argue that traditional social scripts and cultural templates related to gender roles and gender relations are being rendered obsolete by rapidly changing norms [25–27]. Giddens notes that this can be ‘an alien and fragmenting experience’, leaving individuals ‘deskilled’ regarding their abilities to successfully operate in the social world [28]. Young millennial men may be particularly affected by these broad social trends, as evidenced by data from a variety of sources.

For example, much evidence suggests that an increasing number of young men are facing intense difficulties adjusting to adulthood, which is having a detrimental effect on their mental health. This is especially so if a broad definition of mental health is used, such as that suggested by the World Health Organization: “mental health is more than just the absence of mental disorders or disabilities. Mental health is a state of well-being in which an individual realizes his or her own abilities, can cope with the normal stresses of life, can work productively and is able to make a contribution to his or her community” [29].

Statistics from numerous sources indicate that many young men are failing to meet this definition. For example, a 2019 survey found that 29% of millennials always or often felt lonely, and 27% had no close friends, with higher rates in men compared to women [30]. Other studies indicate that boys are performing significantly worse than girls at high-school, with much higher drop-out rates [31–33]. Likewise, surveys indicate that men make up a minority of university students, less than 40% in North America [34] and a majority (over 60%) of people ‘Not in Education, Employment nor Training’ (NEETs) in Canada [35]. Men also have higher rates of drop-out from remedial employment programs for NEETs. [36].

These factors have been identified as social determinants of young men’s mental health and psychosocial well-being, and may contribute towards high rates of suicide, substance misuse and other mental health issues in men [37,38]. Worryingly, evidence suggests that young men under-utilize formal mental health services [39,40], and a recent review indicates that there are few effective or validated programs to improve the mental health and psychosocial well-being of young men [41]. The gravity of this situation has been recognized by many esteemed bodies, including the British Parliament, which launched an official inquiry into the mental health of men and boys in 2019 [42]. Interestingly, participation in the seduction community has not been considered with reference to the growing literature on young men’s mental health and psychosocial well-being. This paucity in the present literature will be redressed in the present study which is uniquely framed in the men’s mental health and well-being literature.

### Aims and objectives

As stated, there is lack of empirical research on the seduction community. The few studies that have occurred have tended to focus on PUA instructors, and there is little social science research on the ordinary rank-and-file young men who participate in the community. To our knowledge, no study has specifically set out to simultaneously understand why men join the community and what kind of impact that participation has on them. It is important to fill this gap in the literature, given the number of men who participate in the community. New research may also contribute towards the growing literature on young men’s mental health and well-being, which is becoming a prominent part of public discourse. As such, the study is propelled by two specific objectives, namely documenting and understanding (i) the reasons that young men join the seduction community; and (ii) the impacts of community involvement on participants’ lives. Mindful of the competing orientations of previous research, the present study attempts to draw a middle-line between a ‘hermeneutic of faith’ and a ‘hermeneutic of suspicion’. We aim to give primacy of voice to participants, allowing them to speak for themselves regarding motives, impacts, perspectives and experiences.

### Epistemological basis

As such, we conducted an *inductive* qualitative study giving ample scope for bottom-up understandings to emerge. Standard procedure in a study that is grounded in an inductive epistemology is to enter the field with a neutral and open mind, eschewing formal hypothesis testing or the employment of a rigid ideologically-driven framework which may bias results [43]. In other words, this is primarily a data-driven rather than a theory-driven study. Thus, the present study is not propelled by theories of masculinity, which has driven much of the aforementioned research on the seduction community [3,5,22]. Instead, the current study takes advantage of the flexibility and openness to surprise that is core to inductively-oriented qualitative research, allowing for fresh perspectives to emerge [44,45]. Moreover, it responds to recent calls for men’s research to move beyond a singular and narrow focus on masculinity, as this may be constraining thought and interpretation on the topic [39,46]. That said, the study proceeded mindful of the literatures and competing orientations previously discussed.

## Methods and materials

### Researcher positionality

The data collection team consisted of the two authors. Both authors were outsiders to the seduction community, and had not been members of the community before the study started. That said, both authors had accumulated much prior knowledge of the community through second-hand consultation of community websites, YouTube Channels and books. This gave them a familiarity with the vocabulary, core concepts and current trends within the seduction community. Both authors are also heterosexual men, with one author being a similar age to most community members (JZ), while another was older (RW)- giving them some level of insider knowledge and status regarding generic men’s issues. All this allowed the authors to occupy the middle ground between insider and outsider status, which has been identified as a desirable position for the rigorous conduct of inductive qualitative research [47]. Indeed, a seminal paper notes that this middle ground has considerable advantages for researchers in terms of ‘building rapport, obtaining ‘emic’ perspectives, maintaining their ethical integrity, and preserving some ‘distance’ for analysis’ [48].

Of note, the first author has decades of experience as a qualitative researcher, and has successfully conducted numerous mental health oriented qualitative studies, including research with immigrant women [49], African-Americans [50], and the rural homeless [51] This experience gave the author many transferable skills which were used throughout the study. As an experienced qualitative researcher, the first author ensured that the study was based on values that have been identified as essential to the ethical conduct of qualitative research including ‘empathy, caring, sharing and understanding’ [48]. Indeed, the study adhered to an influential methodological statement by Dwyer & Buckle that qualitative researchers should primarily be ‘open, authentic, honest, deeply interested in the experience of one’s research participants, and committed to accurately and adequately representing their experience’ [47]. This is the position we adopted.

### Participants

We set out to recruit young men participating in the seduction community, utilizing multiple methods of recruitment. This is a recommended procedure that can help mitigate any selection bias that may be associated with over-reliance on a single method of enrolment, namely by bringing news of the research study to a broad and heterogenous range of potential participants [52,53].

First, the team placed adverts (with contact details) explaining the study in PUA on-line forums and Facebook groups to solicit participation. Second, the team attended PUA ‘lair’ meetings, where they announced details of the study and handed out information sheets soliciting participation. Third, we augmented these methods of recruitment by snowball sampling, where we asked participants to share our contact details with any other individuals in their network involved in the seduction community. All data was collected within Canada from 2017–2018.

Inclusion criteria were as follows (i) attended any seduction community event in the last two years; (ii) engaged in the community on-line, either through regular participation in internet forums or frequent consumption of PUA YouTube or social media channels; (iii) able to give informed consent; (iv) must speak either English or French (v) must be 18 or over. All participants had to meet all criteria. Exclusion criteria were the counterpart of the inclusion criteria. All participants gave written informed consent and the study protocol (number 16/42) was reviewed and approved by the Douglas Hospital Research Ethics Board.

### Procedures: In-depth interviews

All participants were asked to participate in an in-depth semi-structured qualitative interview to discuss their reasons for joining the community and the impact that community involvement had upon them. This qualitative interview was driven by a brief yet structured topic guide revolving around four key open-ended questions (i) why did you become involved in the community? (ii) what are the core aspects of ‘pick-up’ to you? (iii) how and when do you use ‘pick up’? (iv) what impact has community involvement had on your life? Question one was directly related to study objective number one, i.e. documenting and understanding the reasons that young men join the seduction community. Question four was directly linked to study objective number two, i.e. documenting and understanding the impacts of community involvement on participants’ lives. Questions two and three were indirectly linked to the study objectives, allowing us to elicit a more in-depth understanding of young men’s framing and usage of pick-up which could complement responses deriving from the more direct questions. This angular approach is recommended in qualitative research to obtain the desirable thick-description [54,55], and was also essential to our efforts to obtain a holistic understanding of pick-up. These core questions were posed neutrally, however we used follow-up probes throughout. For example, if a participant consistently praised pick-up, we asked if pick-up had any negative effect (or vice versa). We asked for illustrative examples throughout the interviews.

The first five interviews were conducted by the first author with the second author attending and observing. The remaining interviews were conducted by the second author after lengthy training by the first author, including in-situ supervision of his first interviews. All interviews were conducted face-to-face in a place of participants’ choosing. Most interviews were conducted in local cafes, though some occurred in participants’ homes and some in local parks. Participants were given the choice to conduct their interviews in French or English- all chose English. Interviews lasted between one to four hours, and were audio-recorded then transcribed. Additionally, all participants completed a short socio-demographics form before the interview consisting of simple questions eliciting the following: age, education, employment status, housing situation, ethnicity, place of birth, years in Canada, and years in the seduction community. This gave us prompts for follow-up questions during the interview, and allowed us to give basic sample characteristics in the results.

### Procedures: Participant observation

In addition to the interviews, we engaged in lengthy participant observation throughout the project. This follows methodological best practice because (i) participants might give sanitized or socially desirable accounts in verbal interviews; (ii) research indicates that what people say and what they do are often inconsistent; and (iii) it allowed us to triangulate data using two different methods, overcoming biases associated with a single method [56,57].

This participant observation consisted of four key components. First, after each interview, we asked every participant if we could observe them practice ‘game’ for an hour or two, either now or later. This involved an author accompanying (and observing) them as they approached women in parks, stores, clubs, bars or on the streets- debriefing with the participant afterwards. Second, the authors attended various seduction community organized events including ‘lair’ meet-ups and presentations by PUA instructors. Third, an author spent time observing a weekend ‘boot-camp’ where men were being taught by a PUA instructor. Finally, the research team temporarily rented a bedroom in a shared house that was inhabited by a group of active PUAs, spending considerable time with these PUAs in and outside of this house. Copious field-notes were taken during and after these activities. This participant observation deepened the desirable ‘thick description’, complementing the data gathered through in-depth interviews [54,55].

### Analysis

The analysis was based on a commonly used framework known as ‘hybrid thematic analysis’ [58]. This allows analysts to formulate a priori deductive codes, while giving freedom to skillfully create inductive codes. This takes advantage of the flexibility and openness to surprise that is core to qualitative research [44,45]. The semi-structured nature of the interview helped provide an ordered basis for the deductive aspect of the analysis. Super-ordinate deductive codes were based on the questions posed in the interviews, namely (i) reasons for joining the community; (ii) core aspects of pick-up; (iii) uses of pick-up; and (iv) impact of community involvement. Sub-ordinate codes were created inductively, after complete and independent immersion in the data by the two authors, who were joined at the analysis stage by three female research assistants who helped with coding. This immersion included listening to sound files and the in-depth reading of both the interview transcripts as well as the field notes, with regular team meetings to discuss and finalize the emerging coding schema.

Once an incipient coding schema was agreed upon, all transcripts and the field notes were imported into MAXQDA, a computer assisted qualitative data analysis software. A research assistant then coded each interview, allotting paragraphs (or more) of text into the appropriate codes. The field notes were coded analogously, and specifically triangulated for consistency or discrepancy with the verbal interview-based data. Again, the research assistants were closely trained and supervised in this process, including multiple coding of transcripts and field notes; a strong check and balance on validity in qualitative research [59].

In line with standard procedure, the codes were used as building blocks for wider themes [60]. Specifically, at the end of the coding process, we assessed which codes were most prominent and possessed the most explanatory power in relation to both of our study objectives. Overlapping codes were then grouped into wider themes, which form the basis for the results sections below. As is standard procedure in ethnographic research, the qualitative data was not quantified. As such, precise numbers of individuals who mentioned a theme are not given, however a sense of the theme’s significance in the data is provided by use of descriptive language in the text that summarizes the prominence and meaning of the theme.

## Results

The procedures resulted in 34 in-depth interview participants, as well as over 200 hours of participant observation. This sample size is consistent with best practice for a rigorous qualitative study [61,62]. The demographic details of these participants are given in Table 1 below. Of note, over 50% of participants were immigrants to Canada, whom had been in Canada for five years on average. This key fact is revisited throughout the results. Ethnicity was deliberately collapsed into broad categories by the authors to avoid small cells and maintain the confidentiality of participants. Interestingly, both study authors are also immigrants to Canada, which may have increased rapport and trust in these immigrant participants [47,48]. A small number of people were excluded from participation in the study by the study authors. This was mainly because they had only been involved in the community for a very short time, thus lacking the necessary community experience that would enable them to adequately reflect on their involvement.

**Table 1:** Characteristics of sample.

Characteristic	Total number (*n* = 34)
Age in years, mean (SD, range)	25.2 (4.2, 18–37)
% male	100.0
Ethnicity, n (%)	
*European/ Euro-Canadian*	17 (50.0)
*West Asian/North African*	9 (26.5)
*East Asian/ South Asian*	5 (14.7)
*Elsewhere*	3 (8.8)
Place of birth, n (%)	
*Canada*	16 (47.1)
*Outside of Canada*	18 (52.9)
Median time in Canada in years, for those born outside Canada (IQR)	5 (9)
Educational attainment, n (%)	
*High school*	3 (8.8)
*> High school*	31 (91.2)
Employment status, n (%)	
*Employed*	17 (50.0)
*Unemployed*	4 (11.8)
*Student*	13 (38.2)
Living situation, n (%)	
*Alone*	10 (29.4)
*With parent*	8 (23.5)
*With others (e*.*g*., *roommate*, *girlfriend)*	16 (47.1)
% single	61.8
Median time involved in the seduction community in years (IQR)	2 (3)

SD = standard deviation, IQR = interquartile range

The analysis resulted in five overlapping themes. Each theme simultaneously captures common reasons why men join the community, as well as the common impacts of community involvement on participants. These five themes are (i) loneliness and social inclusion; (ii) lack of male role models and need for guidance; (iii) mental health and well-being issues; (iv) skill acquisition and personal development; and (v) the dark side of pick-up. Each theme is discussed in turn below. It should be noted that these themes represent broad patterns in the data, reflecting prominent phenomena that were regularly mentioned by a significant portion of the participants. There were deviant cases for each theme, some of which are presented at the end of each section to communicate the internal heterogeneity within the sample. All quotes are verbatim; however some identifying details have been altered in the text to maintain anonymity and confidentiality.

### Theme one: Loneliness and social inclusion

One of the core themes to emerge from the study was loneliness and its opposite- social inclusion. Many participants reported that they experienced loneliness prior to joining the community. Some reported having few or no friends, consistent with the literature discussed in the introduction. Others reported fractured or problematic relationships with family members. This loneliness could be psychologically painful and functionally impairing. For example, a 24-year-old Euro-Canadian participant was asked why he joined the community. He responded as follows:

I was lonely, I had no friends. I’m a guy who couldn’t talk to a girl sitting next to me in class, forget about going out. But it comes to a certain point where you know, the pain of not doing it, the pain of like being so lonely at 20 years old, I’d just had enough of it, and it was like okay, forget about how painful anything else is…so a friend recommended the book ‘the game’, and then I took action…I went to some free pick-up events and met some wingmen there…

Similar sentiments were expressed by a range of other participants. Interestingly, numerous participants reported that this loneliness negatively affected their health, engendering both frustration and despair. This drove them to get involved in the seduction community. For example, a 33-year-old European immigrant stated that:

When I broke up, I was alone. I was lonely. I had no friends. I had no girls. I had nothing. I'd do my things, my work and everything, but on the weekends, I would pretty much stay in bed all day. I just didn't even feel like getting up. I went through that already once before when I lost all my weight. At a certain point, I said to myself, "This is not the life I want to have. We all have a purpose in life," and I just don't believe that my purpose was to be laying on a couch all day…

Several participants regularly stated that they got involved in the community to address this loneliness and to help in a quest to find a sense of purpose. For many, this meant meeting women and ultimately finding a girlfriend, with one participant simply stating ‘in the beginning, I wanted a girlfriend’ while another stated ‘when I first came to Montreal I’d never dated anyone and I was 25, so I had no idea what I was doing, and to be honest I was still a virgin’. The common desire to meet women to address underlying issues of loneliness and lack of purpose is expressed by a 27-year-old Asian immigrant:

I was a shy person, I had no experience with women…there was a gap there…I decided to get that part of my life settled… I discovered this pick-up program through the internet and I’m like ‘this might actually be interesting!’. I want to have a relationship with a girl, you know, mutual feelings with one another, I think it is better, so that is what I am trying to do…try to be the best version of yourself…focus on higher goals…as a man you need to have your own purpose…

Participants stated that meeting women was a major reason for joining the community. However, they routinely reported that their community involvement was motivated by much more than this. For example, when asked why they joined the community, some participants simply responded ‘to make friends’, both male and female, as evidenced by this 23-year-old Middle-Eastern immigrant:

I was always awkward and lonely all my life. So I was looking, at some point I was like “Fuck! There must be an answer, like fuck!” So I look on the internet…so I went on a pick-up channel and was like, okay I can learn. Like at the time I was not interested in girls at all, and I was a virgin at the time also. So I just want to use that in a way to learn how to make friends, to me I have no idea- I don’t know the process on how to make friends, no books, I didn’t find it at the time. ‘How do I approach people?’ Each time I Google ‘how to approach people’ it was ‘how to approach a girl’. I was like ‘Fuck! I want to learn how to make friend, I don’t care about girl!’

Such sentiments were expressed by others, especially immigrant participants, who often had no friends on their arrival in Canada. Furthermore, such immigrant participants often noted that the cultural norms in Canada were very different from their host countries, leaving them ‘clueless’ (an oft-repeated word) about social dynamics, dating and courtship. This is fleshed out in more detail later in the results.

As stated, a core aim of the study was to understand the impact of community involvement on the young men. Interestingly, participants generally stated that community involvement led to supportive friendships and expanded social networks, leading to a greater sense of social inclusion. For example, one participant, a 23-year-old second-generation immigrant, stated that involvement led to:

A lot of meaningful relationships, I think that is one of the biggest things I have got. I’ve met a lot of interesting people…most of it with guys actually, yea because since you go out with them, you get closer to them.

Such sentiments were repeated by many other participants. Interestingly, participants often focused on the male friends they had made when discussing the impact on their social life. In fact, this helped address the loneliness described by some of the participants. For example, a 29-year-old Euro-Canadian, stated that ‘my social circle has definitely expanded because I am meeting a lot of guys in the group, and a lot of them are really cool guys and we are talking outside of that environment’.

Indeed, we witnessed the communal aspect of involvement first-hand on many occasions during participant observation. This clearly had a positive impact on men who self-described as people who were previously ‘awkward’, ‘lonely’ or ‘clueless’ during the interviews. For example, we attended ‘lair’ meetings, watching in real time as men became mutually acquainted. This is encapsulated in the brief extract from our field notes below, describing one such meeting.

When we arrived, some people were chatting quietly but others were checking their phones alone awkwardly- avoiding any eye contact with others attendees. One man, arriving alone, loudly yet nervously proclaimed his recent luck with ‘Asian women’. He was routinely ignored by the others. The meeting lasted over two hours, and involved several activities, such as introductions, a lecture by two experienced PUAs and practical exercises. This included going onto the street and cheering alone. At the end of the meeting, the change in ambience was palpable. There was a round of applause, and attendees high-fived and hugged each other. People were exchanging contact information and adding each other on Facebook on their phones. No one was excluded, not even me, the oldest man in the room. People then gathered in their own small groups to go out gaming, and invitations were extended to one and all to come along and join in.

To summarize this section thus far, many participants reported joining the community due to loneliness and a lack of purpose in life. Their participation in the community generally resulted in new friendships and an expanded social circle. This led to a greater sense of social inclusion, going some way to address the observed loneliness and help in the young men’s quest for purpose.

That said, not all participants reported that community involvement addressed underlying issues of loneliness and isolation, with some reporting negative social consequences and a negative social ambience in the ‘lairs’. For example, a 23 year old Canadian-born participant stated:

I didn’t really have any social connections [at college]…but I think the community is retarded…like it’s so awful…there is all these guys with really negative ideas…recently there has been a huge uprising of chode…I would never hang out with any of the guys from there now

Similarly, a few participants used the word ‘cult’ when describing the community. For example, one participant stated that ‘I think it’s a cult, but a good cult (laughs)’, while another stated ‘I think it is a little bit culty, I don’t want to get too involved…there is no critical thinking’. One 25 year old Canadian-born participant had mixed-feeling, implying that the community had a strong sense of internal social cohesion that did not extend beyond the boundaries of the in-group, as witnessed in the quote below:

It can be helpful, it can be harmful. I’d say a lot of the time one of the worst things it does is it closes you off to everyone who’s not a pickup person. It’s like you will actually make friends with pickup guys and those will be your only friends because you can’t make female friends because you’re just trying to fuck everyone you talk to, you can’t make any other male friends because they don’t do game; they don’t go out. There are so many guys like that…

Some of the positive aspects related to social inclusion are revisited in theme three ‘mental health and well-being’ below, while some of the negative aspects are expanded upon in theme five ‘the dark-side of pick up’ below.

### Theme two: Lack of male role models and need for guidance

During the course of the study, participants routinely mentioned that they lacked guidance, mentorship and male role models before joining the community. Some intimated that they joined the community to rectify these absences. For example, one simply stated ‘I don't have role models for how to be in good relationships with women’ while another stated ‘I gravitated towards that community because I felt like it offered me a concrete way to fill the gaps’.

Participants routinely reported two ‘gaps’ that needed filling. First, they reported that they lacked generic guidance on ‘how to be a man’; second, they reported lacking specific guidance on how to interact with women. When discussing this topic, participants commonly focused on their parents, often poignantly describing the absence of fathers from their lives due to bereavement, divorce or other factors. For example, a 24-year-old European immigrant stated:

My parents divorced when I was five. I went to live with my mum. I would see my father every second weekend…but I never really talked about that with him so much. And he passed away when I was a teenager, which was a big turning point in my life. I have an older brother…I could talk about it with him, but not super open-mindedly…most of that shit was on my own. I never had any like masculine role model or mentor that would teach me this kind of shit either. I’m self-taught, mostly. I was really emotionally weak and I wasn’t really understanding social dynamics very well before I got into the game.

Indeed, numerous participants noted that they had irregular or limited contact with their fathers when they were growing up. This meant that they were raised by single mothers, who sometimes offered guidance on interacting with women and ‘how to be a man’. Interestingly, many participants reflected on this, stating that this guidance was insufficient and unsuited to the realities of the modern world. For example, a 29-year-old Euro-Canadian stated that:

For example, me, I grew up with a mother and a sister–my father was not there. This happened a lot, too [in the community]. They grew up with their mothers, and their father was not there. My example was a mother and a sister–I remember we were having dinner, and my mother was like, “Oh, yeah…guys, what they should do is to buy flowers for girls,” and stuff like that, and I grew up with this, you know what I mean? So, I was like–my model of the world was shaped by how women think. There–there was a lack of men’s presence when I grew up, and a lot of people in the community, it’s like this. So, kind of made me into, like, a nice guy. And, uh, yeah. I think a lot of people in the community; they just incorporated a lot of woman’s behavior…in psychology there is a theory you learn by watching other people…a lot of them incorporate behaviours from their mother…so their self-image is destroyed, their confidence is destroyed.

The lack of male role models and the negative impact of divorce manifested itself regularly during participant observation. For example we were invited to attend a ‘fight club’ event in a local park with four members of a local ‘lair’, a regular event where men would fight sportingly and then debrief afterwards. The group arrive with boxing gloves, mouthguards, and other fighting paraphernalia. After some small talk, the men expressed a desire to fight some mixed-martial arts, and all are on board, including the researchers. What follows are a series of sporting yet competitive fights, with some slight blood shed and a small black eye to boot. This was the most typically masculine display of raw power we saw in the whole study, yet as we all lay on the grass afterwards the discussion turned to parents. Below is an extract from our field-notes:

We talked about the study, then Brad just started explaining how ‘so many guys are raised by abusive parents, so they are so feminine.’ One of the guys corrected him ‘It doesn’t always have to be abusive, like my parents are good but they divorced when I was like 12’. Brad carried on ‘yea exactly, like me I don’t have a Dad, my mom is abusive as shit.’ I see his eye-brows tightening up with pain as he speaks, a guy who never flinched even when I rocked his head back with a solid upper cut. ‘She was always putting me down like “you ain’t shit!” but hey, a lot of guys, not just me. A lot of guys.’ There was a few seconds of thoughtful silence. Ali changed the subject asking me ‘Where do you get the money to do all this research?’

Relatedly, immigrant participants frequently noted that their fathers grew up in locations outside of Canada and in ‘different times’. Indeed, immigrant participants often reported that their fathers had insufficient knowledge of social dynamics within Canada to help them adjust. Others simply noted that their fathers were busy with other pursuits. In other words, the identified problem was not simply ‘absent fathers’, but ‘old school’ fathers as well as pre-occupied fathers. All this is encapsulated in the quote below, from a 30-year-old European immigrant noting that:

Listen, my parents had me when they were teenagers. Now, my parents are divorced, but each one of them has kids with others. My dad, forget it, never talked to me about anything. My dad was too busy developing and focusing on his own personal life–you know, getting remarried, stuff like that…he wasn't there as much as I'm gonna be there for my kids. My father is old school. I think I could teach him more about women than he could teach me!

The discourse around lack of male role models and need for guidance was a broad pattern observed in the data set, however some participants noted that they had excellent relationships with their fathers and other family members. For example, one participant noted ‘I do have an older cousin that I was especially close with…he was really good at breaking down boundaries…he encouraged me to get out of my comfort zone’. Similarly, a 24 year old second-generation immigrant talked positively about his father, stating that:

My dad. Yeah, he listened to so much of my bullshit, oh my God, all my mistakes, he tries to help me… I respect him a lot for going through what he’s gotten through; changing countries twice, coming here, realizing it’s not so great here when you don’t have money already, doing your maximum…

To end this section, it is worth noting that participants generally concluded that their participation in the community went some way to address their previously unresolved question of ‘how to be a man’. One seasoned member of the community noted that ‘learning game is a lot about integrating male behaviour…you have to hold eye-contact, you have to be confident…it’s about being a man basically’. Another stated that ‘the highest level of understanding of pick-up is internalizing all those high-value behaviours and actions that make you a worthy man, makes you like the man, a fucking man!”. Men commonly reported that this included setting and achieving goals, ‘giving value’ to others and being ‘non-reactive’ to people and events.

That said, some participants reported a potential danger in community involvement vis-à-vis finding male guidance and role models. For example, some participants noted that community members often look-up to PUA instructors and coaches as male role models, sometimes in an unquestioning and uncritical manner. One participant stated that ‘people don’t really question these coaches, that is why it is like a cult (laughs), we basically finance these coaches’ lifestyle’. Such sentiments were reported by another participant, who stated that he travelled across the continent to attend a bootcamp by a popular well-known instructor, noting that ‘bootcamp didn’t help me at all, it was just a waste of money. I paid almost $2000, it was just a waste of time, they are just marketers’. Similarly, another participant referred to some people in the community as follows:

They always look up to the guru. The guru tells them what to do and stuff like this. There is also a monetary incentive from the gurus and instructors you know? Sometimes people take it too seriously “okay, that’s what the leader said, so that’s good. That’s obviously really good” I think we always need to have a critical mindset…you need to take a step back

In sum, some participants joined the community due to a lack of male role models and need for guidance. Community involvement could provide role models and guidance, however participants sometimes reported that there were dangers if this was done in an unquestioning and uncritical fashion.

### Theme three: Mental health and well-being

During the interviews, many participants stated that they suffered from a range of mental health (broadly defined) and well-being issues before joining the community. Some had been diagnosed with a discrete mental illness, but the most common could be labelled as ‘well-being’ issues that can constrain psychosocial functioning, but are not officially recognized as a mental illness. The most common issues raised revolved around the related concepts of shyness, social anxiety and introversion; concepts that reappeared throughout the dataset. Many participants reported suffering from these issues, and addressing them was a prime motivation for community involvement. For example, a 28-year-old Euro-Canadian stated:

In my case, it was more–like, in high school, for example, I was, like–I had a little bit of friends, but it was more, like, social anxiety always. I was always, like, socially anxious. I remember, like, some girls were, like, interesting to me, and I was just, like, too shy to, like, have a girlfriend and walk with her. I was just super anxious, I don’t know why.

Interestingly, numerous participants raised the concepts of shyness and social anxiety in answer to the very first question of the interview ‘why did you get involved with the community?’. One participant stated ‘between 18 and 20 –I was a shy person. Well, basically, in high school, I was a shy person. So, I had almost no experience with women, and that kind of threw me off’. Another stated that ‘I didn’t like who I was. I wanted to change who I was in regards to women, you know? I was, you know, shy, um, very like uncertain of myself, you know?’ Similarly, a 24-year-old Euro-Canadian answered the first question of the interview as follows:

Because, well, I started this a few years ago and I’m 24 now. So I was like the super shy kid in high school; no friends, prior to getting in this community I’d never been on a date, never had a kiss, never held a girl’s hand, nothing, I was terrified of women, a real lack of knowledge.

Another related concept that arose throughout the interviews was that of ‘social awkwardness’, with participants noting that this summed up a self-assessed lack of social skills and communication skills. One immigrant participant stated that ‘I was always awkward and lonely all my life. So I was looking, at some point I was like “Fuck! There must be an answer!”. Another Middle-Eastern 23 year old immigrant noted:

I’m kinda, let’s say socially awkward I would say. I have always been like an introvert as well, I wasn’t like that super funny guy who can just make the crowd laugh and everything. So, I guess that’s something that I was missing in my life. Yeah, and I just decided to, to take care of it and this is how I started.

Similarly, some participants stated that they were a ‘nerd’ prior to joining the community, and that this contributed to social awkwardness, anxiety and shyness. Again, this had a negative social effect, and was a prime motivator for community involvement. One participant stated that before joining the community ‘I was kinda the nerdy kid, I was much more introverted, I was much more self-conscious, but I had the feeling of missing out’. A 32-year-old Asian immigrant noted that:

I met people but I was still too scared to go outside. I was like the cliché computer nerd who plays video games. I understand the nerd language (laughs). So I was just getting frustrated, so last year I decided ‘Okay it is time for me to go out and take action’

As stated, most of the issues raised by these participants were related to concepts such as awkwardness and shyness, which do not constitute official mental illnesses and are typically considered personality traits or generic well-being issues. That said, a minority of participants noted that they had experienced clinical mental illnesses which had negatively affected their lives. This sometimes prompted them to join the community as part of their recovery. For example one participant stated approvingly that ‘my self-esteem really improved, because I was depressed before I found fitness, and then fitness brought me up to a certain level, but pickup brought me up way over that!’, while another noted that ‘I do think that, really, I was depressed for the longest time’. The depth of this issue is witnessed below, from a 30-year-old immigrant:

I started to get a bit depressed. Start doing a bit of drinking–quite heavily, actually–doing a bit of cocaine, gaining weight. Just wasn't being able to get out of bed. I was depressed for maybe five years, so I didn’t work, didn’t pay rent, I was getting thrown out from place to place. All I wanted to do was lay down at home and do nothing and eat double cheeseburgers. I felt myself sliding in a slope and I said ‘fuck no, it’s not gonna happen’

As seen above, some participants reported that they joined the community to address a range of issues related to mental health and well-being. This begs the question- what impact did community participation have on these issues? In short, participants commonly stated that participation had a positive effect. In general, participants noted that community involvement successfully addressed issues of shyness, social anxiety and awkwardness, as witnessed in the quote below from a 25-year-old Euro-Canadian participant:

I think I'm much less guarded, less shy, more open. I feel less inwardly focused, more expansive, and less thinking about "everybody's looking at me" and stuff like that. I don't care. What else? Being part of this community is kind of making new acquaintances, new people that I hang out with. The first year I was in this city, I didn't have many friends. Now, I think I have more abilities to relate to people and have fun times with people.

The positive impact on shyness was witnessed continuously by the research team during participant observation. For example, after each qualitative interview, we asked participants if they would allow us to watch them approach a random stranger and debrief afterwards. Almost all participants excitedly acceded to our request, confidently striding up to random women and starting a conversation. Interestingly, almost all of the women approached engaged positively with the participant, and well over half of the interactions led to an exchange of phone numbers or Facebook details. This is encapsulated in a quote from a 32-year-old immigrant, that summarizes the positive effects experienced by some participants:

My life sort of took a 360 –even a 720 if you could. I signed up to the gym. I have lots of new friends, people who actually care. They call, "How are you?" Not just girls–guys and people I meet at work. I would say it is a complete 360. I’m a completely different person now. I don’t have anxiety anymore, I don’t feel depressed. I am friendlier, I am happier, I’m more willing to help other people. I’m no longer the pathetic guy that sits around and says ‘why me?’ I don't know. I don't know what I would do without it.

It should be reiterated that this theme represents a broad pattern in the data. Not all participants reported mental health and well-being issues prior to joining the community. By the same token, not all participants reported universally positive benefits as a consequence of community involvement. For example a 30 year old North African immigrant reported that:

I think it can impact both positively and negatively. Well, if it helps you, like, build a relationship with people, a positive–I think, for sure, it can help, but, uh, sometimes, uh, it also makes you very nervous, especially when you get bad rejections, it hurts, really, your feelings and this can be also a very negative, you know. And sometimes for–I think it depends on the people. For some sensitive people it just–it has more impact, I would say, on them than, like, others.

Indeed, other participants reported negative impacts on various dimensions of well-being. These are fleshed out in more detail in theme five ‘the dark side of pick-up’.

### Theme four: Skill acquisition and personal development

To summarize the results so far, participants generally reported that they experienced a range of painful psychosocial issues before joining the community. This included loneliness, lack of guidance and mental health/ well-being issues including shyness and social anxiety. It is therefore unsurprising that analysis revealed that many participants noted that they joined the community to acquire new and valued social and communication skills. Participants hoped that these skills could help address the issues described in the previous sections, and better still help them flourish and thrive within Canadian society. For example, a 29-year-old Asian immigrant noted that:

So back in Asia, uhm, I, I like I um, see myself as an introvert. So I don’t really enjoy talking to people that much. And back in my country, um I had a normal life and I didn't feel the need to actually go approach people and try to make friend that much. But when I moved here, there was like a big change of culture and, like for 6 months, um I couldn’t talk to people and I couldn’t really connect with people that much. That’s why um, I went on YouTube –… and looked for, yeah, how to pick up girls, how to talk to women and stuff. So I found out um, one of those channels… so suddenly my mind there was like a switch, and um, I figured out “okay this is possible to go on street and talk to strangers.” Sooo, then after that I tried it, and it worked, and I was happy…

Indeed, immigrant participants in particular noted that they arrived in Canada lacking the social and communication skills that are functionally valuable in North American society. These immigrants noted that they came from countries with different cultural scripts and templates governing male-female relations and social relations per se. This void hindered their abilities to interact with women, as well as general social integration and inclusion within Canada. In fact, a word commonly used by immigrant participants was ‘clueless’, when discussing their attempts at social integration in Canada. For example, one participant, a 23-year-old Middle-Eastern immigrant, noted that:

I was really clueless about how to make this stuff happen and I feel like a lot of uhh, of people our age, umm, you know it’s like the generation of broken men. You know like, I was raised by a single mom so I never really learned, I never had a fatherly figure to show me what it was like to be a man and how a man is supposed to interact with women properly when they’re young…and yeah like I mean my whole life growing up I was clue—, clueless with social skills, I mean I had so much trouble making friends cuz I switched countries when I was younger and whatnot. I was raised in fucking rural Lebanon so obviously culturally it’s completely different you know. And then they moved me to Canada and I had to learn to live with completely new people…the whole culture shock, I was getting bullied, having trouble meeting people…I had no guidance. It took me three years to make my first friend…and, then I was like ‘oh my god! This is a skill, I can learn it!’, I learned many skills in my life before so let’s just do it! So I figured out about the game and I was like ‘let’s look at this free method and learn some techniques!’

As seen above, the perceived lack of culturally-appropriate skills hampered some participants’ abilities to make friends, meet women and generally operate optimally within Canadian society. This led some of them to get involved in the seduction community, which participants noted has considerable free content and resources devoted to skill acquisition available on YouTube. Many participants devoutly consumed this material and reported that it has a positive affect on skill acquisition. For example, a 22-year-old Euro-Canadian participant stated:

When I started college my social skills were back at zero and my social skills went up to the point where I am now comfortable going into a bar and I can own the room just because I am so confident in my ability to do so. Pick-up gave me the motivation to learn a lot of new skills. Like right now I’m learning to speak Arabic just because my girlfriend is Tunisian so I’m learning to speak Tunisian Arabic. I’ve learned to play keyboard. I read a lot now. I engage a lot socially. So, all of these different skill sets have all been impacted just because I’ve thought to myself I should be a more interesting person, so I just picked up little things here and there and I learned to do them to just like an intermediate level where I can impress.

Another participant noted the positive impact on social skills, with one stating that ‘I landed quite a few job interviews by networking and applying my pick-up thoughts and theories and experience’. In addition to these social skills participants reported that their involvement in the community equipped them with valuable communication skills, with one participant stating that:

Since I have been learning about pick-up…from what I have heard from others, I give very excellent presentations today in comparison to high school where I’d be staring at the paper, hands shaking, monotone voice. Now I really control the room, and I love it!

Improvements in public speaking were reported by many other participants. One stated that ‘it helped me develop my confidence in front of people when I am talking’, while another said ‘I would not have made it to management consultant without pick-up’. Indeed, participants regularly reported that the skills they acquired had a global impact on their lives, with many purposely noting that this extended beyond sex and seduction. For example, one participant emphatically noted that ‘everything you learn from it is applicable everywhere else…so I think of it as just a super good social training…it totally extends beyond sex’, while another stated ‘it’s a process that helps you in all aspects of your life, in the end it helps for everything, it’s really complete- it’s not just for the girls’.

The importance of skill acquisition and its global impact on personal development was conveyed to the research team many times during participant observations. This is neatly summarized in the following interaction. One afternoon, the two authors were walking with one of the respected veteran members of the community. He had encouraged us to learn pick-up ourselves, as had many participants, stating repeatedly that these were useful skills for all. At one point, he encouraged us to make conversation with a woman ambling slowly on a local street, who was wearing sunglasses and headphones, with advice on ‘how to open’. We hesitated for many reasons, but decided that such activity was an integral part of our methodology. As such, an author approached the woman, trying to follow the advice: a confident pose, strong eye contact, a smile and friendly opening words. The interaction met with success, and the women removed her headphones, sunglasses and a long conversation ensued. At the end, she stated that ‘I’m really glad you came to say hi…can I add you on Facebook?’ Our field notes record the following:

On my way home, I pondered on how I approached the woman, and why it worked. I felt a new energy, a new confidence and joy that filled me for no particular reason. A sense of accomplishment came to me, I think it is because I have just done something that I have never done and never thought I would do before. I can see how this may be addictive.

To close this section, it is worth noting that this theme represents a broad pattern in the data, and not every participant reported skill acquisition as a core motive for joining. For example, one participant noted that ‘For me, growing up, I didn’t have a lot of the problems of the people in the community. I didn’t necessarily have the fear of talking to people because that always came pretty naturally to me’. Similarly, a 24 year old South American immigrant stated that:

For me, I’ve always kind of been a natural when it came to talking to people. For me, I have always been able to make people comfortable. Because I had the social skills. I always did. I just learned little things [in the community]

### Theme five: The dark side of pick-up

So far, the analysis indicates that participants mainly became involved in the community in a self-directed effort to address personal psychosocial deficits such as loneliness, shyness and awkwardness. Participants generally reported that community involvement helped address these deficits. That said, participants sometimes reported that community involvement could have a negative impact, as already witnessed in some of the quotes presented in some of the above sections. As participants regularly discussed a dark-side to pick-up, this is presented as a theme in its own right, given its importance and prominence in the data-set.

To start, some participants reported that they became ‘addicted’ to using pick-up to meet women; spending large amounts of time and energy engaging in game-related activities. Some reported that the ensuing lifestyle negatively interfered with employment and education. For example a 24-year-old unemployed Middle-Eastern immigrant stated that:

I was totally-addicted. It was soul-eating. I discovered I was addicted about 3 or 4 years in, and I realized that my life was taking a drastic turn. I was in school but I wasn’t interested in school anymore. I was heavily into running but I wasn’t interested in a single bit of running. I was just interested in clubs and girls…and I was tired of just focusing on girls and not focusing on my life…I decided to build myself first, that is what I am doing now…it’s dangerous if you are not ready!

Indeed, others noted that their participation in the community negatively influenced their studies. A graduate student stated that ‘it has kind of negatively affected my research…sometimes I stay awake till 4am doing some crazy stuff, and that can affect your day after so it’s not good really’. Such sentiments were repeated by others who were in graduate school or professional occupations, even those who felt the community had mainly benefited their personal development. For example, a 23-year-old immigrant graduate student noted:

This had a really positive effect in my professional life, in my social life, outside of girls and in my self-confidence…but the thing with PUA is that it takes a lot of time, it did take a toll on my studies at university, because you are constantly thinking about it…you are walking to class and you are like ‘how can I approach her?’ In class you do not focus, you are looking at this girl. So I’m super happy I got out of it. Get out of it once you’ve reached that potential, it’s diminishing returns after that.

This potential to interfere with daily functioning frequently came up during participant observation, with our field notes reporting one participant stating that ‘he had some problems because he allowed it to take over his life’ while another noted ‘how disruptive it had been for his work, and how time consuming the whole thing is’. Throughout the study, participants mainly stated that they thrived in the community, but that they saw other men struggle, with one participant stating that ‘he saw many younger guys harmed by pick-up and is saddened by it’. Similarly, a 22-year-old Euro-Canadian participant stated:

I think it can be healthy, but it can also be unhealthy, and can turn into maybe even a problem, an addiction for some people. I think it is an interesting set of tools and mindsets, but if you look at some of the guys who have been doing it for a long time, they can’t stay in long term relationships. I think that is a sort of testament to the way it manifests long-term.

Occasionally, participants reported witnessing very serious issues, with one stating that a local member ‘practices, practices, practices, but still has no results. He is so bitter and depressed that sometimes he becomes violent when other guys come into his set. At one point he wanted to commit suicide’. Importantly, the issue of violence was not a theme in the dataset, and we did not witness any acts of violence during the participant observation. However it should be noted that one of the self-reported darker aspects of pick-up reported by some participants was its negative psychological effect on people around them, especially women. One participant noted that ‘It's kind of weird referring to girls as "sets" and stuff like that. I think it's also kind of objectifying’. Another 24-year-old African immigrant had recently disengaged from the community, outlining his reasons for doing so during an interview:

At the end I saw women as like objects; I saw women as like treats, I saw women as like nothing really, like nothing. I dismissed completely their emotions. That’s why I stopped; I didn’t continue, yep. That’s why I didn’t continue, because I realized if I continued I’d be like wrecking some girls, you know. I didn’t want to do that.

These sentiments of guilt and shame, deriving from an emerging awareness that their actions might be harming some women, were shared by other participants, who built on this theme, either by reflecting on their own behaviour or the behaviour of others in the community. For example, another participant noted that ‘I think there’s a number of people who just use it [the community] to pump their own egos and make money from it’, while another lamented men who join the community ‘to just fuck a bunch of girls and pick-up girls and then treat them like shit- that’s fucking selfish’. These comments imply that some men in the community mobilize their own needs as a means to achieve sex, increases in confidence or other outcomes, without consideration of the well-being of the women being ‘gamed’.

Interestingly, some others additionally reflected that these types of ‘selfish’ extreme pick-up behaviours can damage more than the women being gamed, but also one’s own self-respect and sense of moral rectitude. For example, a 24-year-old Euro-Canadian participant noted that:

But again, I’ve seen it have the effect on some people where game teaches that men are competition and women are objects, you know. It objectifies women, demonizes men. I have definitely gone through both of those phases. Some of them just manipulate everyone they talk to; it’s not even just women, it’s game everyone, game everyone. I know guys that will pretty much sleep with anything with a vagina just because lay count, lay count, lay count; they’re not even attracted to her but they just want to be able to say, ‘I fucked this many girls’, right? I mean honestly I went through a phase where I was not open to dating women at all; I just wanted to go in and fuck.

Other participants reported that such promiscuity was damaging to their own mental health. Two concepts that regularly arose in this regard was ‘the soul’ and ‘emptiness’. Both of these concepts are used by a 29-year-old Asian graduate student who had recently got into a relationship and disengaged from pick-up. Of note, this participant casually uses the derogatory word ‘bitch’ to refer to women, which was sometimes used in a similar manner by other participants:

I see myself as an introvert, and when I moved here there was a big change of culture and I couldn’t really connect with people…so that’s the main reason I was doing this…then I tried it, and it worked, and I was happy, but then I just wanted to fuck as many bitches as I can, but I could feel like its empty.…during one week, I was with 4 or 5 different girls but the next day I felt this emptiness, and I would say each of them take a part of your soul, and make you more empty…because of that I stopped doing that…and I knew this was a mentally healthy thing to do

A similar narrative was reported by other participants who had recently disengaged from the community. They stated that they joined the community to learn needed social skills and address psychosocial deficits. They successfully learnt these new skills which improved many areas of their lives, including success with women. However, they disengaged from the community after periods of promiscuity, sometimes when starting a more serious relationship. For example, a 25-year-old immigrant reports:

The reason I stopped essentially is because I met this girl I really liked, and I was really like ‘oh why am I doing this?’ There’s no purpose and I’m just like draining a part of my soul. Because, each, each girl you meet and have sex with, it’s like if you’re giving her part of your soul. And, at some point it’s like if you broke your soul into like, a lot of fragments and you’re giving it away and you like feel empty because you’re not, you don’t have this special relationship with anyone.

Indeed, participants regularly reflected on the harmful aspects of promiscuity, as witnessed in an interaction with a participant as we were preparing for a qualitative interview. This participant confessed that he often lied while ‘gaming’ women, developing a ‘fake front’ for these interactions. The field notes report that this participant arrived for the interview jocular and confident, but the mood momentarily changed, as witnessed in the extract below:

He read the consent form and chuckled at some of the academic phrases…he jokes about the café looking like someone’s living room…he then asks me if I had a girlfriend, and I told him I am married. He then asks how old I am and how my wife and I met- which I answered honestly. He looked down in thought silently then said “you are a nice man…not like me…you are a nice man…I have a girlfriend and I lie to get with other people all the time…you are a nice man”. For the first time, he avoided my gaze and resumed reading the consent forms contemplatively.

Again, it should be stated that not every participant reported a dark-side to pick-up. That said, the transcripts commonly included a complex mix of positive and negative reflections on pick-up and involvement in the seduction community. This is encapsulated in the words of a 28 year old Canadian born participant, who ended his interview by summarizing these mixed-feelings. This is an apposite extract to close the results:

Anything you do, there's negatives, I guess. But in my book, the positive outweighs the negative by far, you know? Yeah, I guess I see some negative. You can take anything and turn it into negative, you know? But I feel like, in this day and age, this movement, this energy, is positive. If I had to put a label on it, it's positive. Is it perfect? No, but it's generally a positive upward spiral kind of vibe for me. [laughs]

## Discussion

This study aimed to better understand why men join the seduction community, as well as what kind of impacts community involvement has on them. The results indicate that there is much nuance and complexity in answer to both these questions, suggesting that unidimensional understandings of men in the community as merely desiring sex and ‘success with women’ are simplistic and superficial. To deepen understandings, the three most prominent findings of the study are interpreted and situated within the appropriate social science and mental health literatures below.

### Why join the community?

The first key finding of this study is that participants often join the seduction community in an attempt to address a range of psychosocial deficits that are impeding their ability to flourish and thrive. These include struggles with a range of mental health and well-being issues including loneliness, isolation, social awkwardness, shyness and lack of male guidance. Additionally, immigrant participants reported difficulties with social and cultural integration due to differing social norms and cultural scripts in Canada.

This finding overlaps with some of the existing literature on the seduction community discussed in the introduction. In particular, Schuurmans & Monaghan found that men joined the seduction community to address issues of loneliness and isolation [6]. Moreover, these authors also found that a disproportionate number of men in the community were immigrants struggling with issues of social integration. This finding is consistent with a wider body of research indicating high rates of loneliness and isolation among young millennial men [30,63], as well as many other psychosocial difficulties in the transition to adulthood [31–36].

The findings regarding the lack of male role models (particularly fathers) and need for guidance overlap with much existing literature. For example, the marriage rate in Canada is currently at its lowest ever, and rates of divorce are reaching over forty percent, meaning more and more young people are being raised by a single parent [64]. Importantly, statistics from the Department of Justice indicate that mothers are awarded exclusive custody of children in almost 80% of cases in Canada, with fathers often having minimal amounts of time with their children [65]. Of note, much research indicates that father-absence can have a detrimental effect on the social development, mental health and psychosocial well-being of growing children, especially boys [31,32,66]. This was witnessed in the present study, with participants often lamenting the absence of involved fathers or other male role models that could assist in their psychosocial development.

Similarly, the finding that men join the community to address mental health and well-being issues is consistent with research indicating that men under-utilize formal mental health and well-being services, preferring informal action-based interventions to formal talk-based interventions [38–40]. Indeed, one way of interpreting the results is that men are using the seduction community as an unofficial mental health and well-being service, as part of a quest to address ongoing psychosocial issues. Such an interpretation is consistent with other research indicating that men tend to avoid formal mental health services due to common perceptions that they are inherently ‘feminised’ and unresponsive to men’s needs [67,68].

### What is the impact?

The second key finding is that participants generally reported that their involvement in the community led to a range of psychosocial benefits that improved their mental health and well-being. The communal involvement led to new friendships and social support from fellow PUAs, and their participation did indeed make them feel part of a much-needed community. This indicates that the appellation ‘seduction community’ is not a misnomer and that communal benefits are reported by participants. These findings are divergent from O’Neill [3] who notes ‘the term ‘community’ is also something of a misnomer…concealing the machination of…a lucrative industry’. Of course, framing these men as members of a community serves a functional purpose for those who trade off and commercially profit from these programs, as this may make program engagement more appealing. That said, communal benefits were reported by many participants, indicating that involvement had socially functional purposes for the men themselves.

Similarly, participants generally reported that they gained valuable new social and communication skills within the community, both vertically (from instructors) and horizontally (from peers). These were used productively in the dating world, but also in other domains including employment. Participants tended to report that all this increased their self-confidence, self-esteem and overall psychosocial functioning, successfully addressing prior psychosocial deficits.

This finding overlaps with some of the results from the previous ethnographies of the seduction community. For example, Hendriks notes that community teachings and practices sharply focus on various aspects of self-help beyond ‘success with women’ including self-discipline, self-improvement, personal growth and individual empowerment [4]. Likewise, O’Neill notes that participation in the community often led to an improvement in self-confidence and social skills, leading to professional benefits [3].

Interestingly, this finding is also consistent with Giddens’ seminal work on ‘skilling’, which was discussed in the introduction [28]. As noted, leading sociologists cogently argue that rapidly changing social norms can strip people of the ability to successfully operate in the social world [25–27]. Giddens calls this process ‘deskilling’ [28]. Though participants did not use Giddens’ terminology of ‘deskilling’, many used the analogous term ‘clueless’ in reference to their previous selves. They reported using the seduction community as a venue to ‘reskill’, with some success. This overlaps with other sociological scholarship indicating that informal self-help communities are common arenas of ‘reskilling’ [69].

### The dark-side of pick-up

The third key finding is that participants reported a dark side to their involvement in the community, which could take various forms. Some noted that they had become addicted to community involvement, which negatively interfered with their daily functioning including education and employment. Others noted that their involvement led to a promiscuous lifestyle, which could become deeply unsatisfying and damaging to their ‘soul’. Some reported that it led them to objectify and manipulate women and other men, while we occasionally heard derogatory language towards women and men in the interviews.

These findings vis-à-vis the objectification of women overlap with some of the literature discussed in the introduction. For example, a content analysis of books written by PUA instructors concludes that their writings are ‘characterized by…the objectification of women…in compliance with hegemonic masculinity’ [5]. Likewise, O’Neill notes that the instruction techniques used by PUA instructors mean that ‘women are not only objectified, but made into object lessons’ [3]. The participants in the current study reported that this objectification did occur, and that this could be harmful to women, as well as to the men themselves.

Interestingly, other studies also report that involvement in pick-up can have a detrimental effect on the men involved. For example, Schuurmans and Monaghan similarly found that community involvement can have ‘an all consuming nature…and costs in terms of time, money and men’s general well-being’ [6]. Likewise, Hendriks notes that ‘members of the seduction community not only objectify women, but also other men, and they objectify themselves’ [4]. We witnessed similar phenomena in our results.

### Mental health and well-being implications

The findings from the current study have implications for young men’s mental health, especially where mental health is broadly conceptualized in line with the WHO definition given in the introduction [29]. As stated, participants generally joined the community to address a range of psychosocial deficits. Participants routinely reported that the community successfully equipped them with a range of life skills which they used effectively in areas such as employment and education. Participants highly valued the peer support, the guidance from other men and the acquisition of skills which led to personal development. As such, the community appeared to fill a void in providing a place of hope, social learning and fellowship for young (often immigrant) men.

As previously stated, men tend to under-utilize formal mental health and well-being services, sometimes perceiving such services as overly ‘feminized’ [39,67,68]. Of note, a recent review found a dearth of evidence-based programs to improve young men’s psychosocial well-being [41]. All this implies the need for tailored mental-health and well-being services for young men. Could anything be learnt from the practices of the seduction community in designing such new services?

To be sure, participants reported a dark side to the seduction community, which can damage the men involved, and the women with whom they come into contact. This means that blindly instructing men to join such groups, or wholly reproducing them, may be inadvisable. However, several of the activities reported within the results incorporate effective aspects of mental health promotion. Perhaps these could be distilled and used as key ingredients for innovative new programs that aim to improve young men’s well-being.

Most notably, some of the practices common within the seduction community have some resemblance to the various aspects of Cognitive Behavioral Therapy (CBT)- one of the most effective transdiagnostic mental health interventions [70]. For example, CBT therapists encourage patients to acquire new psychosocial skills, setting goals in an ‘action-plan’ that includes considerable ‘homework’ to help them reach these goals [71]. For a common mental issue like anxiety, ‘homework’ may involve initiating conversations with strangers, or ‘cognitive restructuring’ which involves identifying and correcting maladaptive self-evaluations [72].

Interestingly, participants frequently reported activities such as goal setting, action-plans and ‘homework’ that they learnt and practiced within the community. They learnt this through (i) on-line and in-person PUA courses; (ii) consultation of PUA instructor YouTube channels; and (iii) on-line and in-person peer support from other members of the ‘lair’. This peer support included giving and receiving social, emotional and instrumental support based on demographic affinity and ‘expertise by experience’- thus resembling more formal mental health peer support [73]. Again, participants did not use CBT terminology, but instead used a parallel but related PUA vocabulary such as learning and practicing a ‘cold approach’ (i.e. initiating a conversation with a stranger) or working on their ‘inner game’ (akin to cognitive restructuring).

One approach to improving young men’s mental health could be the creation of similar male-led and male-focused group interventions. These could offer similar on-line and in-person activities focused on skill acquisition, personal development and mentoring- minus the harmful practices and emphasis on sex and seduction. Such initiatives could be offered in educational institutions, thus making them a structural meso-level intervention, perhaps helping to address some of the wider issues previously discussed, including low rates of school and university completion among young men [31–33]. As well as imparting skills and catalyzing personal development, such groups could also discuss and address other issues raised in the present study including social isolation, lack of acculturation and wider gender issues- with an emphasis on positive pro-social solutions. Such interventions would be consistent with the conclusion of a recent systematic review of young men’s well-being programs, which stated that ‘male targeted interventions may be more beneficial for young men than gender neutral programs’ [41].

### Limitations of the study

This study has a number of limitations. First, it is a purely qualitative study, that did not involve enumeration or the use of objective before and after measures vis-à-vis mental health and well-being. A quantitative longitudinal approach would give more precise knowledge about the impact of the seduction community on young men. That said, the current study points to emerging patterns and raises hypothesis for future research.

Second, we mainly recruited from PUA forums, websites and inner-circle meetings. This may have led to some level of selection bias, inasmuch as men who are disenchanted with the community may not have been adequately reached. As such, we are not making any claims that theoretical saturation was reached and we did note a degree of internal heterogeneity in the sample, which is presented in the results. Thus, further research with larger samples may be necessary to access and gain the full perspectives of current and former members of the community, especially those with a more negative experience, which may lead to other themes and perspectives.

Third, we did not collect data from women who had come into contact with members of the seduction community, as the aim of the study was to better understand the men involved. Eliciting the perspectives of such women should be a future research priority, and would allow for the better triangulation of data to fully understand the wider impact of pick-up on women and society as a whole.

Fourth, it may be that participants gave socially desirable responses which masked their authentic beliefs and motives, especially as the seduction community has been stigmatized and condemned by the mainstream media in recent years [15,17–21]. For example, it may be more socially acceptable to cite loneliness as a reason to participate in the community, rather than cite a desire for a hedonistic or promiscuous lifestyle which is often associated with sexual predation and sexual immorality. In other words, participants might be narrativizing their accounts on the basis of what they have learnt within the community, sensitive to wider societal concerns and stigmas. This is hard to ascertain in the present study, and such concerns may be overcome through more anonymous methods such as completion of on-line nameless questionnaires.

Fifth, researcher positionality may have affected participant responses, perhaps contributing to socially desirable responses. For example, both of the authors are outsiders to the seduction community, and we were always honest and transparent about this position. Consistent with qualitative best practice, we attempted to collect data in an empathic and non-judgmental manner [47,48], however our outsider status may have deterred some participants from complete openness during the interviews. Relatedly, both the study authors are heterosexual men who are immigrants to Canada, and therefore had some degree of demographic and phenomenological affinity with the life experience of participants. This may have unconsciously shaped some of the findings, which are somewhat less hostile and suspicious than research conducted by people lacking this affinity with seduction community participants [3,22] That said, both authors maintained the recommended degree of analytical distance from the participants during the study, as witnessed by the fact that neither of us are still in contact with any participants. Moreover, much of the coding was conducted by female research assistants who lacked such affinity, which may have acted as a check and balance on the risk of the two authors ‘going native’ during the analytical and interpretive process.

## Conclusion

The ordinary rank and file men within the seduction community have been demonized by sections of the media, largely due to the activities of a few prominent PUA instructors. However, the present study uncovers much nuance and complexity in the reasons why men join the seduction community, as well as the impacts that involvement has on them. Most significantly, the study reveals that men join the community to address a range of psychosocial deficits, and that community involvement helps equip participants with a range of valued social and communication skills. This seemed to be especially beneficial for immigrant participants, who are overrepresented in the community. That said, there is a dark side to community involvement.

Nature abhors a vacuum. As such, it may be that young men are joining the seduction community in the absence of alternative male-focused and male-led tailored supports that can help them make the transition to adulthood. Thus, there is a pressing need for new and tailored initiatives (and accompanying research) to improve the mental health and well-being of young men, which can incorporate some of the male friendly approaches identified in this paper, minus the harmful practices and the emphasis on sex and seduction. Such efforts may help address the oft-ignored psychosocial problems plaguing a growing number of young men in our society.
